# Cell-Penetrating Peptide-Mediated Therapeutic Molecule Delivery into the Central Nervous System

**DOI:** 10.2174/1570159X11311020006

**Published:** 2013-03

**Authors:** Li-Li Zou, Jie-Lan Ma, Tao Wang, Tang-Bin Yang, Chang-Bai Liu

**Affiliations:** 1 The Institute of Molecular Biology, Medical School of China Three Gorges University, 8 Daxue Road, Yichang 443002, China; 2 Key Laboratory for Pathogenic Microorganism, Medical School of China Three Gorges University, 8 Daxue Road, Yichang 443002, China

**Keywords:** Central nervous system, blood-brain barrier, cell-penetrating peptides, drug delivery.

## Abstract

The blood-brain barrier (BBB), a dynamic and complex barrier formed by endothelial cells, can impede the entry of unwanted substances – pathogens and therapeutic molecules alike – into the central nervous system (CNS) from the blood circulation. Taking into account the fact that CNS-related diseases are the largest and fastest growing unmet medical concern, many potential protein- and nucleic acid-based medicines have been developed for therapeutic purposes. However, due to their poor ability to cross the BBB and the plasma membrane, the above-mentioned bio-macromolecules have limited use in treating neurological diseases. Finding effective, safe, and convenient ways to deliver therapeutic molecules into the CNS is thus urgently required. In recent decades, much effort has been expended in the development of drug delivery technologies, of which cell-penetrating peptides (CPPs) have the most promising potential. The present review covers the latest advances in CPP delivery technology, and provides an update on their use in CNS-targeted drug delivery.

## INTRODUCTION

The blood-brain barrier (BBB) is a dynamic and complex barrier that exists in all vertebrate organisms with well-developed central nervous systems (CNSs) and which is interposed between the blood and CNS to protect the brain against invading pathogenic organisms [1, 2]. It is also the most unique and challenging barrier formed by endothelial cells to impede the entry of unwanted substances such as therapeutic molecules into the CNS *via *the blood circulation [3, 4]. As the number of people who suffer from CNS-related diseases increases, there is an urgent demand for the development of effective therapeutic methods to enable therapeutic molecules to cross the BBB [5].

Despite rapid developments in our understanding of the BBB and great advances in medical technology, many CNS-associated diseases remain beyond the reach of conventional therapies [6]. This predicament is not caused by a lack of candidate therapeutic molecules but by the failure of nearly 98% of small molecules and 100% of large molecules to cross the BBB into the CNS [7, 8]. The only way to overcome this obstacle is to find a suitable delivery system. As the existing delivery methods may conflict with the natural functioning of the BBB, the ongoing effective approaches should be carefully studied with respect to their influence on the overall protective function of BBB. Considering the poor efficiency of the traditional delivery methods, the present review just covers the aggressive research efforts that are being made in this field, paying a close attention to the development of new cell-penetrating peptide-based therapeutic strategies.

## THE STRUCTURE AND PHYSIOLOGICAL FUNCTION OF BBB

The BBB, as a continuous impermeable cellular barrier, is primarily composed of non-fenestrated brain endothelial cells characterized by tight junctions (TJs), which dramatically limit the transport of unwanted substances to the CNS [9]. TJs at the BBB are composed of an intricate combination of trans-membrane proteins and cytoplasmic accessory proteins, which are linked to the actin cytoskeleton to form extremely restrictive cell-to-cell connections [1, 3]. The structural properties of TJs, such as the diffusional features enforced by the lipid bilayer and the directional characteristics of the specific transport proteins in the cell membrane make the BBB a continuous cell membrane [4, 6]. Furthermore, the TJs act as a fence in the lateral cell membrane that segregates unwanted substances to either the luminal or abluminal membrane domain and impose restrictions on their free movement from one side of the endothelium to the other [10].

In addition, it is now well accepted that the functional structure of the BBB includes more than just brain microvessel endothelial cells [11], and several other cell types such as adjoining pericytes and astrocytes are the critical determinants that maintain the brain capillary phenotype. These cells combine with the extracellular basal membrane and microglia to form the support system of the BBB [10, 12, 13]. Together with the surrounding neurons, these components form an intact and functional neurovascular unit [14]. Brain endothelium, the principal and most effective part of the BBB, is formed from abundant exchange vessels, such as capillaries and post-capillary venules, with thin walls and wide surface scales. In the human brain, approximately 100 billion capillaries make up a total length of approximately 650 km and a total global surface area 20 m^2 ^of brain capillary endothelium [15].

Therefore, the unique biological characteristics of the BBB owe its low permeability to a variety of important factors, including: (1) very few pinocytotic vesicles [2, 16, 17]; (2) TJs strengthened by the interaction of astrocytes and pericytes with brain endothelia cells [18]; (3) the synergistic inductive functions of certain cells, such as astrocytes, pericytes, perivascular macrophages and neurons [19-22]; (4) the permeability restriction of unwanted agents by ATP-binding cassette (ABC) transporters, insulin receptors, multidrug resistance-associated proteins (MRPs), and transferrin receptors [23-25]; and (5) the lack of lymphatic drainage and the absence of the major histocompatibility complex (MHC) [26]. All these features of the BBB make it a selective barrier, which, in conjunction with the cerebrospinal fluid (CSF), continuously flush therapeutic molecules back to the blood stream [27, 28].

## THE THERAPEUTIC MOLECULE DELIVERY TECHNOLOGIES FOR CROSSING THE BBB

### Passive Diffusion

Passive diffusion, a concentration-dependent transport method, allows multiple lipid-soluble therapeutic molecules through the BBB without an energy requirement [29, 30]. There is high correlation between the crossing rate of therapeutic molecules and their lipid solubility and molecular weight [31, 32]. Lipid solubility can be determined by the logD octanol/water partition co-efficient at pH 7.4 [33] and only the molecules with a molecular weight less than 400 Da are permeable to the BBB [34]. The co-effect of lipid solubility and molecular weight is believed to be attributable to the functional expression of several ABC transporters that deliver therapeutic molecules across the BBB [35]. However, these are not always the absolute restriction factors for BBB penetration. Currently there are many types of efficient CNS-active drugs in clinical use, such as those drugs that carry a positive charge. Basically, their entry is probably due to the reaction between the positively charged drugs and the negatively charged glycocalyx and phospholipid of the outer leaﬂet of the BBB [36]. For example, lipophilic compounds such as hydroxyzine and triprolidine can access the CNS by passive diffusion when administered *via *the nasal cavity [37], and acetylcholinesterase reactivators also appear to undergo passive transport through the BBB [38].

### Endogenous Carrier-Mediated Transports

Endogenous carrier-mediated transport, which involves ATP- and transporter-dependent active transport, solute carrier (SLC) transporters, and receptor-mediated endocytosis, is characterized by selectivity and saturability and requires energy expenditure by the cell [30, 39]. For ATP-dependent active transport, breast cancer resistance protein (BCRP), P-glycoprotein (P-gp) and the MRP family are the classical transporters [39]. BCRP is a multidrug resistance protein that mediates apically directed drug transport [40]. Prazosin and cimetidine are two typical substrates of BCRP [41]. P-gp in brain capillary endothelial cells functions as an efflux pump in the physiological state and P-gp-mediated efflux of cyclosporin A is a major reason for its restricted transfer from the blood to the brain [42]. MRP proteins contribute to the cellular efflux of endogenous anionic glutathione or glucuronate conjugates (substrates for MRP1), cyclic nucleotides (substrates for MRP4 and MRP5), and glutathione (co-substrate for MRP1 and MRP4) [43]. Although transporting a variety of substrates across the BBB into the CNS by BCRP, P-gp and MRP has been widely described, low efficiency is still a major problem for these endogenous carrier-mediated transports [39, 44-49].

The substrates for BCRP, which is expressed at the luminal membrane of the BBB, include cytotoxic compounds, sulfated conjugates of therapeutic drugs, and hormones; the overlapping profile and similar localization of those substrates demonstrate that the BCRP has a limited BBB penetration [44]. P-gp, a member of the ABC transporter superfamily, is also expressed at the luminal membrane of the BBB and can positively pump a variety of therapeutic drugs back into systemic circulation [45]. Recent study suggested that the cooperation of P-gp and BCRP can transport substrates from endothelium to blood [46]. The MRP family, comprised of nine members (MRP1-9), is competent efflux pumps that are capable of the delivery of structurally diverse lipophilic anions [47]. Some members of the MRP family seem to be located in either the luminal or the abluminal membrane, or sometimes both [48]. For example, MRP1, MRP4, and MRP5 are clearly localized on the luminal side of brain capillary endothelial cells, MRP4 and MRP5 have also been detected in astrocytes of the subcortical white matter, and MRP5 is present in pyramidal neurons [43]. Together, they may play a role in the chemoresistance of the BBB [49].

Most polar molecules cannot passively diffuse through the BBB and the cells in the BBB express a large number of SLCs [44]. There are 51 families of SLC transporters and they play crucial roles in numerous cellular physiological processes, including importing or exporting nutrients, neurotransmitters and metabolites [50]. Among the members of the SLC family, the organic cation transport (OCT) system (SLC21) and organic anion transport (OAT) system (SLC22) are of particular interest due to their roles in delivering therapeutic molecules across the BBB. Depending on the sub-cellular localization of these transporters at the BBB, endogenous therapeutic molecules can be transported into or pumped out of the brain [39].

Some therapeutic molecules usually can be delivered across the BBB *via *the receptor-mediated transcytosis (RMT) system, which is generally a three-step procedure involving receptor-mediated endocytosis at the blood side followed by intracellular movement and exocytosis at the brain side of brain endothelial cells [15]. Several receptors on the BBB, such as the transferrin receptor (TfR), insulin receptor (IR), IGF receptors 1 and 2 (IGFR1 and 2), the low-density lipoprotein receptor (LDLR), the low-density lipoprotein receptor-related proteins 1 and 2 (LRP1 and 2), the scavenger receptors class A type I (SR-AI), class B type I (SR-BI) and diphtheria toxin receptor, have been extensively studied as part of the RMT system [51, 52]. RMT allows large molecules to be transported across the BBB and is thus a useful method for the delivery of peptides, proteins and certain peptidomimetic monoclonal antibodies into the brain [22, 53]. For example, insulin and transferrin are transported from blood to brain by IRs and TfRs, respectively [54].

## DELIVERY OF THERAPEUTIC MOLECULES ACROSS THE BBB MEDIATED BY CELL-PENETRATING PEPTIDES

Although multi-disciplinary approaches are now available, they still can not meet the needs of high doses while limiting the risk of major side effects. Thus, molecule delivery systems that cross the BBB constitute a major piece of the therapeutic puzzle, and new efficient therapeutic molecule delivery systems are still urgently required. Recent rapid advances in molecular biology have enabled the development of novel delivery systems that take advantage of our better understanding of the BBB. Compared with the majority of delivery systems that suffer from various limitations when applied in clinical situations to the transport of therapeutic molecules into the CNS, cell-penetrating peptide (CPP)-based delivery systems show a great ability in carrying macromolecules across cellular membranes, combining a low cellular toxicity with high efficiency [55]. Considering their smaller size (up to 30 amino acids in length), cationic and/or amphipathic CPPs have a greater potential to penetrate the BBB than other transport systems, enabling them to be used as very promising tools for therapeutic purposes in CNS-related diseases [56]. 

The first CPP, trans-activator of transcription (TAT), derived from human immunodeficiency virus-1 (HIV-1), can be efficiently taken up from the surrounding media [55, 56]. Another penetratin, also known as Antp, derived from the third helix of the antennapedia transcription factor of *Drosophila melanogaster *[57], is, together with TAT, regarded to be the first group of CPPs derived from natural proteins. Since then, the number of CPPs with effective transduction properties has greatly expanded. The second group of CPPs consists of chimeric molecules, such as transportan (TP), which consists of 12 amino acids derived from the neuropeptide galanin fused with a 14 amino acid peptide from the wasp venom mastoparan [58]. The third group of CPPs consists of the synthetic peptide family, of which polyarginines are the best studied [59, 60].

**Fig. (1):**
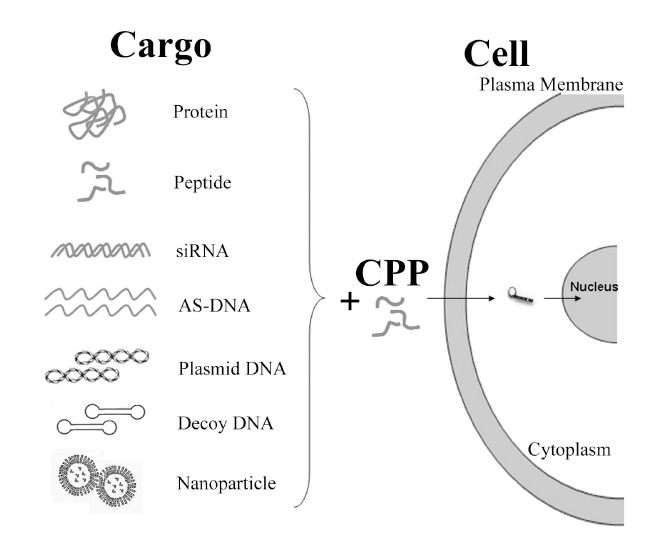
Applications of CPPs in cargo delivery.

CPPs are of different sizes and amino acid sequences but with one distinct feature, which is capable to deliver various cargo molecules across the plasma membrane，some even can cross BBB to facilitate the delivery of various therapeutic molecules; thus, they act as molecular delivery vehicles (Fig. **1**) [60]. A number of CPPs have already shown great ability in improving therapeutic molecule delivery across the BBB to treat CNS diseases [61]. The CPPs used include TAT, Angiopep, penetratin, TP, rabies virus glycoprotein (RVG), prion peptide, and SynB [62-67]. Different CPPs use distinct cellular translocation pathways, which depend on cell types and cargos, even though the delivery mechanisms are still under debate [60]. Generally, there are two types of mechanisms now proposed: direct penetration and endocytosis-mediated entry. Direct penetration of CPPs occurs *via *an energy-independent cellular process and it is believed that translocation across biological membranes can progress at 4^o^C and most likely involves a direct electrostatic interaction with negatively charged phospholipids [68]. Recently, a detailed model for direct penetration has been proposed, which involves strong interactions between CPPs and the phosphate groups on both sides of the lipid bilayer, the translocation of charged residues across the hydrophobic core of the membrane and the passive diffusion of these highly charged peptides across the membrane through the formation of aqueous toroidal pores. This mechanism explains how key ingredients, such as the cooperativity between the peptides, the large positive charge, and specifically the guanidinium groups, contribute to uptake [69].

Endocytosis-mediated entry is the process of cellular ingestion, where the plasma membrane folds inward to bring substances into the cell. During this process cells absorb substances from outside of the cell by imbibing it with their cell membrane. Study has shown that the cellular entry of penetratin by endocytosis is an energy-dependent process, and that this process is initiated by polyarginines interacting with heparan sulfates that promote endocytosis [70]. Research has also shown that TAT is internalized through a form of endocytosis called macropinocytosis [71]. Another endocytosis-mediated entry is based on the formation of inverted micelles. Inverted micelles are aggregates of colloidal surfactants in which the polar groups are concentrated in the interior and the lipophilic groups extend outward into the solvent. According to this model, a penetratin dimer combines with the negatively charged phospholipids, thus generating an inverted micelle inside of the lipid bilayer. The structure of the inverted micelles permits the peptide to remain in a hydrophilic environment [72, 73].

In summary, CPPs hold great potential as *in vitro* and *in vivo* delivery vectors for research and clinical use and they have been successfully used for the delivery of therapeutic molecules such as proteins, peptides, nucleic acids, small molecules and nanoparticles into cells.

### Delivery of Proteins and Peptides Across the BBB by CPPs

Therapeutic protein and peptide delivery across the BBB by traditional cargo methods has been challenged by the multiple limitations offered by the complicated physiological surroundings. Although gene therapy is an attractive treatment strategy for many diseases, the therapeutic use of viral vectors in some acute diseases such as acute stroke is not feasible because the delayed increase in active protein levels in patients after transfection result in little benefit. An alternative way to deliver even larger proteins is to use CPPs, a quick, simple and safe delivery system. It occurs in a concentration-dependent fashion and enters into cells through the lipid bilayer component of the cellular membrane. In recent studies, several CPPs have been used for the delivery of biologically active peptides or full-length proteins into the CNS [74] (Tables **1**-**2**). Several studies have shown CPP-mediated delivery of fusion proteins *in vitro* [74-75]. However, only a few studies have used CPPs as the vehicle to traffic the peptides or proteins across the BBB into the CNS. The first evidence for CPP *in vivo* delivery was provided by Schwarze *et al*. [76]. They confirmed that β-galactosidase fused with TAT can be transported into almost all tissues, including the brain, when intraperitoneally injected into mice.

Cao *et al*. [77] injected a TAT-Bcl-xL fusion protein into the stroke mouse model, and found that the mortality of neuronal cell death was greatly decreased in the area of ischemic damage. TAT-Bcl-xL treatment before and after ischemia can also reduce infarct volume and neurological deficits after prolonged ischemic insults lasting 90 min. Furthermore, reduced cerebral ischemic damage and protection against ischemia in brain injury have also been reported by using TAT-neuroglobin, TAT-islet-brain-1 (IB-1)/JNK-interacting protein-1 (JIP-1), TAT-PSD-95, TAT-NR2B9c peptide and TAT-GDNF [73, 78-80].

Fu *et al*. [81] have developed a vehicle based on a 39-amino acid peptide derived from rabies virus glycoprotein (RDP) to efficiently target β-galactosidase and brain-derived neurotrophic factor (BDNF) to the CNS by systemic administration of RDP-fusion proteins. Furthermore, the RDP-BDNF fusion protein showed neuroprotective properties including a reduction in stroke volume and neural deficits in mouse stroke models [82]. Jo *et al*. [83] used a CPP composed of a hydrophobic signal sequence derived from fibroblast growth factor 4 (FGF4) to deliver the physiological inhibitor suppressor of cytokine signaling 3 (SOCS3) to the brain. The results implied that replenishing the intracellular stores of conditionally labile SOCS3 with cell-penetrating forms of SOCS3 can effectively suppress the devastating effects of acute inflammation.

### Delivery of Nucleic Acids Across the BBB by CPPs

Transfection of cultured cells with nucleic acids (e.g., plasmids, decoy oligodeoxynucleotides, siRNA, and antisense oligonucleotides) are well described, and provide for primary neurons, especially for brain neuronal cells *in vivo*, a high-yield method that is distinct from that provided by viral vectors. Viral transfection has certain drawbacks, including the complexity of vector preparation, safety concerns, and the generation of immune or inflammatory responses *in vivo*. Meanwhile, the main problem of non-viral nucleic acids transfection methods is a low efficiency when compared with viral vectors. To overcome these problems, the CPP-nucleic acid conjugate method has been developed, which is proving to be a very powerful tool (Tables **1**-**2**).

As genes can be inserted into the compatible sites of plasmids, and the corresponding complexes can be transfected into living cells, plasmids, which have had a seminal role in the advances of genetic engineering, have been identified as a promising therapeutic approach. A method that uses macro-branched TAT has been proposed for plasmid DNA delivery into various mammalian cell lines and has shown significant transfection capabilities [84]. Furthermore, multimers of TAT have been found to increase the transfection efficiency of plasmid DNA by 6-8 times more than poly-L-arginine or mutant TAT2-M1 and by 390 times compared with standard vectors [85]. Knight *et al.* [86] reported that when the nontoxic fragment C (HC) of tetanus toxin is covalently bound to polylysine [poly(K)] it can improve the binding of DNA and lead to high transfection efficiency *in vitro* in N18 RE 105 cells (a neuroblastoma X glioma mouse/rat hybrid cell line) and F98 cell (a glioma cell line).

Lo *et al.* [87] has observed that the transfection efficiency of DNA in human and rat glioma cell lines was enhanced 7,000-fold when plasmid DNA was mixed with TAT with 10 linked histidine residues (TAT-10H) instead of the original TAT; a further increase in efficiency was found if two cysteine residues were incorporated into the TAT-10H peptide. For gene transfection of CNS cells, CPP-modified nanocarriers, such as TAT-modified liposomes or micelles that allow for intracellular delivery, may be a better way [88]. Researchers always use polyethylene glycol (PEG)/polyethylenimine (PEI)-shielding to improve particle pharmacokinetic behavior, a targeting ligand to facilitate particle-cell recognition and in some case a bioresponsive lipid or pH-triggered polymer to enhance nucleic acid release and intracellular trafficking [89]. A number of groups have observed that a CPP-modified nanocarrier can be an effective system for plasmid delivery. For example, PEI/DNA-TAT improves the cellular uptake of gene vectors and enhances the gene transfection efficiency of primary neurons up to 14-fold [90]. However, CPP-modified nanocarriers require more *in vivo* studies to support the development of appropriate clinical applications in CNS disease therapy.

Distinct from plasmid DNA delivery, the CPP-mediated transfection of cells with small interfering RNA (siRNA) *in vitro*, and even *in vivo*, has been extensively studied [91-93]. RNA interference (RNAi) is a naturally occurring gene silencing process whereby cells degrade complementary mRNA molecules that may be a promising therapeutic approach for many CNS diseases, including brain tumors, neurotrauma, neuropsychiatric diseases, neuromuscular diseases, pain, and infections [94-96]. The BBB is essentially impenetrable to any RNA molecule and part of the reason is that the average mass of any possible siRNA molecule will be approximately 14 kDa. Most animal models and preclinical studies on the delivery of siRNA molecules to the brain are through invasive routes so there is an urgent demand to develop new delivery methods to improve the efficiency of therapeutic siRNA through the systemic route across the BBB. 

The rabies virus can enter into neurons because the RVG interacts specifically with the nicotinic acetylcholine receptor on neuronal cells. Kumar *et al.* [66] showed that RVG-9R can deliver therapeutic siRNA to target neuronal cells *in vivo*, providing 80% protection against fatal viral encephalitis in mice after three days treatment. Alvarez-Erviti *et al. *[97] reported that siRNAs were successfully delivered to the mouse brain by systemic injection of RVG-modified exosomes. This study demonstrated that the mRNA and protein levels of beta-amyloid precursor protein (APP) cleaving enzyme 1 (BACE1), a therapeutic target in Alzheimer’s disease, were knocked down by as much as 60% and 62%, respectively, by RNAi in wild-type mice. Hwang *et al.* [98] also delivered siRNA and microRNA to the mouse brain using an RVG-modified PEI nanocarrier (RVG-PEI-SS) as vehicle.

Other CPPs also can be used as therapeutic siRNA delivery systems. For example, Davidson* et al.* [99] has found that penetratin can deliver siRNA duplexes to primary mammalian hippocampal and sympathetic neurons *in vitro* and to the CNS in an *in vivo* rat model when coupled with siRNA through a thiol-based linkage. This approach caused less toxicity towards the targeted cells in comparison with the liposome-based siRNA approach.

Abundant human genetic diseases caused by mutations can lead to aberrant alternative splicing. Pre-mRNA splicing correction is another promising therapy for these kinds of diseases. Antisense morpholino oligonucleotides (AMOs), a type of neutral single-stranded DNA derivative with a morpholine ring instead of a sugar moiety, complementarily bind to a target point and correct the pre-mRNA splicing by adjusting the splicing localization, thereby providing a potential therapeutic tool for genetic diseases [100, 101]. However, this strategy has been limited by low correction efficiency *in vivo* and the inability of AMOs to cross the BBB efficiently. Due to their membrane translocation properties, CPPs covalently coupled to an oligonucleotide can increase the delivery of these kinds of molecules, delivering them directly into the cytoplasm and ultimately the nucleus. For example, (RXRRBR)_2_XB, an arginine-rich CPP (R=L-arginine, X=6-aminohexanoic acid, B=beat-alanine), can dramatically improve ataxia-telangiectasia mutated (ATM) splicing correction efficiency when conjugated with specific AMO [101]. The systemic administration of the FITC-labeled (RXRRBR)_2_XB-AMO showed efficient uptake by the brain of model mice.

Besides siRNA and splicing correction, CPPs can also be employed to deliver other nucleic acids, such as antisense oligonucleotides, peptide nucleic acids (PNAs), and decoy DNA. Caille *et al.* [102] have demonstrated that penetratin-APP antisense oligonucleotides reduced APP expression and embryonic neural stem cell proliferation in the subventricular zone (SVZ) of the CNS in adult mice. Decoy DNA, consisting of exogenous double-stranded DNA (dsDNA), was designed to imitate the promoter sequence to inactivate specific transcription factor activity [103], but its poor bioavailability has always hindered its usefulness as a therapeutic nucleic acid. Fisher *et al*. [104] confirmed that the conjugation of TP/TP10-PNA hexamer or nonamer with NFкB decoy DNA can efficiently depress interleukin-1-induced NFкB activation and interleukin-6 gene expression. 

### Delivery of Small Molecule Drugs Across the BBB by CPPs

CPPs have also been exploited for improving the transport of small molecules (e.g. chemotherapeutic drugs) across the BBB (Tables **1**-**2**). In addition, TAT-modified micelles have been used to improve the delivery of small molecule drugs, like ciprofloxacin, across the BBB to the brain [105, 106]. SynB peptides and Angiopeps are the most extensively studied vehicles for the delivery of such drugs to the brain [107].

Conjugation of drugs to members of the SynB family of peptides has been shown to increase their activity in the brain *in vivo* [108]. Rousselle *et al*. [109] reported that the brain penetration of a variety of poor brain-penetrating drugs, including doxorubicin, benzylpenicillin, paclitaxel and dalargin, was significantly enhanced when the drugs were conjugated to SynB1 or SynB3 and injected intravenously into mice. Drin *et al*. [110] reported that SynB1 and SynB5 significantly increase the uptake of doxorubicin into the brain. De Boer *et al*. [52] found that SynB3 enhanced the brain uptake of chemotherapeutic agents both in *in situ* brain perfusion and in an *in vitro* BBB/cell model, and further study in a clinical trial showed enhanced transport of morphine-glucuronide to the brain. Furthermore, Rousselle *et al*. [111] have increased doxorubicin transport into the rat brain by up to 30-fold by covalently coupling the drug to two peptides, SynB1 and penetratin.

Angiopeps, a family of Kunitz domain-derived peptides, have also been used in an *in vitro* BBB model and *in vivo* studies to deliver drugs to the brain with a high efficiency. Demeule *et al*. [112] reported that Angiopep-2 can transport Alexa 488 labeled paclitaxel across the BBB to treat brain cancer. ANG1007, the Angiopep-5 conjugated to three doxorubicin molecules, killed cancer cell lines *in vitro *and crossed the BBB with a dramatically high influx rate [113]. In recent years, much effort has been made to use Angiopeps to deliver drugs or nanoparticles across the BBB to the CNS, showing that Angiopep-mediated targeting is one of the most promising ways to reach the CNS for treatment of brain cancer or brain metastases [114-116].

### Delivery of Nanoparticles by CPPs

Nanoparticles, sized between 1 and 100 nanometers, can, in terms of their properties, behave as a whole unit. TAT was recently harnessed by Santra *et al*. [117] to penetrate the BBB barrier and deliver CdS: Mn/ZnS quantum dots (Qdots) into rat brain tissue. Histological results clearly showed that TAT-Qdots migrated beyond the endothelial cell line of injection to reach the brain parenchyma [118]. Liu *et al*. [119] provided compelling evidence that TAT facilitates *in vitro* human brain endothelium cell uptake of nanoparticles self-assembled from TAT-PEG-β-cholesterol and that, more importantly, the nanoparticles with TAT modification were able to cross the BBB and translocate to the nucleus of neurons. Wang *et al*. [120] studied TAT-cholesterol-G(3)R(6) in a *C. neoformansme* meningitis rabbit model, which revealed that these nanoparticles crossed the BBB and showed antimicrobial activity against the pathological strains in the brain tissue. Qin *et al*. [121] found that the majority of TAT-modified liposome (TAT-LIP) accumulated in the brain within 24 h after administration *via *tail vein injection, even though not all of them were selectively targeted to the brain. These studies hold importance for TAT-mediated transport of nanoparticles into the brain for treatment of brain infections and tracking of nanoparticles *in vivo*, bringing us a step closer to the development of clinically applicable nanocarriers for the treatment, as well as monitoring, of CNS-related diseases. In addition to TAT, other CPPs also have successfully been employed for drug delivery in brain [122, 123]. For example, Angiopep-2, conjugated to polyamidoamine (PAMAM) *via *bifunctional PEG and then complexed with the DNA, designated as PAMAM-PEG-Angiopep/DNA nanoparticles, can be a potential delivery system for gene therapy of glial tumor [124] (Table **2**).

## CONCLUSIONS

An efficient and safe way to treat CNS disorders by systemic drug application is still lacking, despite the considerable effort that has been expended in the development of efficient and reliable drug-carrier systems. CPPs, which are an attractive type of therapeutic molecule delivery vehicle due to their low toxicity and the wide variety of cell types that they are capable of targeting, represent a potentially valuable tool for the intracellular delivery of therapeutic molecules to alter intracellular signaling pathways as well as interfere with intracellular interactions to rebalance a perturbed cellular function and protect neurons in cases of ischemia and neurodegenerative diseases. Preclinical studies demonstrating the successful use of CPPs indicate that CPPs possess great clinical potential for treating various CNS-related diseases by transporting therapeutic molecules across the BBB. Although CPPs have proved to be very useful tools to promote the delivery of therapeutic molecules across the BBB, yet the poor status of potent CPPs available in customized conditions produces a dismal picture for further clinical application. It is armed with this improved knowledge that scientific interest in the field has risen but a lot needs to be done. Hoping drug delivery aimed at the CNS can move into a more passionate phase.

## Figures and Tables

**Table 1.** Applications of Representative CPPs *in vivo*

**Applications of Representative CPPs in Proteins Delivery**				
**CPPs**	**Sequences**	**Cargo**	**Target**	**Summary**
TAT	YGRKKRRQRRR	β-Gal/GDNF/ JNKI1/PSD-95	Brain	β-Gal fused to TAT resulting in strong β-Gal enzyme activity in mice brain; TAT-JNKI1 administration 3 hours after brain ischemia significantly reduced the infarct volume; TAT-PSD-95 protected cultured neurons from excite-toxicity, and reduced focal ischemic brain damage; TAT-GDNF protected brain neurons from cell death when administered after focal cerebral ischemia [76, 79, 80]
TAT-HA	YGRKKRRQRRR-YPYDVPDVA	Bcl-xL	Brain	Administration of TAT-HA-Bcl-xL into mice decreased cerebral infarction in a dose-dependent manner, as determined at 3 d after 90 min of focal ischemia [77]
RDP	KSVRTWNEIIPSKGCLRVGGRCHPHVNGGGRRRRRRRRR	BDNF/β-Gal/Luc	Brain	RDP-BDNF showed the neuro-protective properties in mouse experimental stroke including reduction of stroke volume and neural deficit; The brain slices with X-Gal staining were determined the delivery of RDP-β-Gal across the BBB; The time-course relationship of RDP-Luc was studied to confirm the transport efficiency of RDP [81, 82]
FGF4	AAVLLPVLLAAP	SOCS3	Brain	FGF4-SOCS3 protected mice from lethal effects of staphylococcal enterotoxin B and lipopolysaccharide by reducing production of inflammatory cytokines and hemorrhagic necrosis brain [83]
**Applications of Representative CPPs in Nucleic Acids Delivery**				
**CPPs**	**Sequences**	**Cargo**	**Target**	**Summary**
TAT-10H	5H-YGRKKRRQRRR-5H	Plasmid DNA	Brain	5H-TAT-5H/DNA complexes improve up to 7000-fold in BBB across efficiency over the original Tat peptide [87]
RVG-9R	YTIWMPENPRPGTPCDIFTNSRGKRASNG-9R	GAPDH/BACE1 siRNA	Brain	RVG-9R delivered GAPDH/BACE1 siRNA to neurons, microglia, oligodendrocytes in the brain, resulting in a specific gene knockdown [97]
Penetratin	RQIKIWFQNRRMKWKK	APP antisense oligonucleotides	Brain	Penetratin-APP antisense oligonucleotides reduced APP expression and embryonic neural stem cell proliferation in the subventricular zone of the CNS [102]
**Applications of Representative CPPs in Small-Molecule Drugs Delivery**				
**CPPs**	**Sequences**	**Cargo**	**Target**	**Summary**
SynB3	RRLSYSRRRF	Morphine-glucuronide	Brain	Enhanced the brain uptake of morphine-glucuronide in in situ brain perfusion [52]
SynB1/SynB3	RGGRLSYSRRRFSTSTGR/RRLSYSRRRF	Dalargin/Paclitaxel	Brain	Enhanced dalargin in brain uptake, resulting in a significant improvement of anti-nociceptive effect [109]
SynB1/SynB5	RGGRLSYSRRRFSTSTGR/RGGRLAYLRRRWAVLGR	Doxorubicin	Brain	Significantly increase the uptake of doxorubicin into the brain [110]
SynB1/D-Penetratin	RGGRLSYSRRRFSTSTGR/RQIKIWFQNRRMKWKK	Doxorubicin	Brain	20-fold increase of doxorubicin in brain parenchyma by using a capillary depletion method, [111]
Angiopep-2	PFFYGGSGGNRNNYLREEY	Paclitaxel	Brain	Angiopep-2-paclitaxel showed activity in heavily pretreated patients with brain metastases and/or failed prior taxane therapy [115]
Angiopep-5	RFFYGGSRGKRNNFRTEEY	Doxorubicin	Brain	Angiopep-5-doxorubicin exhibited dramatically higher BBB influx rate constants than doxorubicin and pooled within brain parenchymal tissue [113]
**Applications of Representative CPPs in Nanoparticles Delivery**				
**CPPs**	**Sequences**	**Cargo**	**Target**	**Summary**
TAT	YGRKKRRQRRR	Qdots	Brain	TAT-Qdot was loading sufficiently high in brain that a gross fluorescent can be visualized using a low power UV lamp [117]
TAT-Liposome	YGRKKRRQRRR- Liposome	Coumarin-6	Brain	TAT-LIP was a promising brain drug delivery system due to its high delivery efficiency across the BBB [121]
TAT-cholesterol	YGRKKRRQRRR -cholesterol	G(3)R(6)	Brain	FITC-loaded cholesterol-conjugated G(3)R(6)-TAT can cross BBB, and is a promising antimicrobial agent for treatment of brain infections caused by *C. neoformans* [120]
TAT-PEG-cholesterol	YGRKKRRQRRR-PEG -cholesterol	Ciprofloxacin	Brain	TAT-PEG-cholesterol were effective for delivery of ciprofloxacin across the BBB [119]
RVG-BPEI-SS	YTIWMPENPRPGTPCDIFTNSRGKRASNG-BPEI-SS	cy5.5-miR-124a	Brain	The RVG combined with BPEI-SS for neuron-specific targeting *in vivo* is sufficient to deliver neurogenic microRNA (e.g. cy5.5-miR-124a) into the brain [98]
(RXRRBR)2XB	RXRRBRRXRRBRXB	AMO	Brain	Systemic administration of (RXRRBR)2XB-AMO in mice showed efficient uptake in the brain, which can dramatically improve ATM splicing correction efficiency [101]
Angiopep-2-PEG	PFFYGGSGGNRNNYLREEY-PEG	Paclitaxel -Alexa488	Brain	Fluorescent microscopy revealed that Angiopep-2 modified PEG nanoparticle deliver Alexa488 labeled paclitaxel across BBB, and localized in brain endothelial cell monolayers [112]
Angiopep-2-O-MWNTs-PEG	PFFYGGSGGNRNNYLREEY-O-MWNTs-PEG	Doxorubicin	Brain	Fluorescence imaging demonstrated that Angiopep-2-O-MWNTs-PEG constituted an ideal dual-targeting drug delivery system to deliver doxorubicin for the treatment of brain tumor [114]

**Table 2.** Recent Applications of Representative CPPs *in vitro*

**Applications of Representative CPPs in Proteins Delivery**				
**CPPs**	**Sequences**	**Cargo**	**Target**	**Summary**
TAT	YGRKKRRQRRR	JNKI	Neurons	TAT-JNKI administration 6 hours after oxygen glucose deprivation reduced neurons death at 24 hours [78]
**Applications of Representative CPPs in Nucleic Acids Delivery**				
**Name**	**Sequences**	**Cargo**	**Target**	**Summary**
TAT	YGRKKRRQRRR	Plasmid DNA	Various cell lines	Confocal imaging showed that TAT-DNA mediated transfection was 3-fold more efficient than a standard PEI transfection [85]
HC-[poly(K)]	QYIKANSKFIGITEL-poly(K)	Plasmid DNA	Neuroblastoma / glioma mouse/rat hybrid cell line	HC-[poly(K)]-DNA, resulting in non-viral gene delivery and marker gene expression *in vitro*, was dependent on HC and was neuronal cell type specific [86]
RVG-9R	YTIWMPENPRPGTPCDIFTNSRGKRASNG-9R	FvE siRNA	Neuronal cells	RVG-9R delivered FvE siRNA to the neuronal cells, resulting in specific gene silencing within the brain, and afforded robust protection against fatal viral encephalitis in mice [66]
Penetratin	RQIKIWFQNRRMKWKK	Caspase-3/8/9 siRNA, SOD1-1/2 siRNA	Primary mammalian hippocampal and sympathetic neurons	Penetratin-caspase-3i/8i/9i siRNA can be uptake by primary mammalian hippocampal and sympathetic neurons efficiently; Penetratin-thiol linker-deliver SOD1-1/2 siRNA to neurons simply, efficiently, and without toxicity [99]
TP/TP10	GWTLNSAGYLLGKINLKALAALAKKIL/AGYLLGKINLKALAALAKKIL	NFкB decoy DNA	Various cell lines	Conjugation of TP/TP10-PNA hexamer or nonamer with NFкB decoy DNA can efficiently depress interleukin-1-induced NFкB activation and interleukin-6 gene expression [104]
**Applications of Representative CPPs in Small-Molecule Drugs Delivery**				
**CPPs**	**Sequences**	**Cargo**	**Target**	**Summary**
Angiopep-5	RFFYGGSRGKRNNFRTEEY	Doxorubicin	Brain cancer cells	Angiopep-5-doxorubicin killed brain cancer cell lines *in vitro* [113]
SynB3	RRLSYSRRRF	Morphine-glucuronide	Brain cancer cells	SynB3 enhanced the brain uptake of morphine-glucuronide through an *in vitro* BBB model [52]
**Applications of Representative CPPs in Nanoparticles Delivery**				
**CPPs**	**Sequences**	**Cargo**	**Target**	**Summary**
TAT-PEG-Cholesterol	YGRKKRRQRRR- PEG-Cholesterol	FITC	Brain astrocytes cells	Confocal laser scanning microscopy reveals that the uptake of FITC-TAT-PEG-Cholesterol by human astrocytes was much higher than that of free FITC [119]
PAMAM-PEG- Angiopep-2	PFFYGGSGGNRNNYLREEY	Plasmid DNA	Brain glial cells	PAMAM-PEG-DNA-Angiopep-2 can be a potential delivery system for gene therapy of glial tumor [124]
PEI-TAT	YGRKKRRQRRR-PEI	Plasmid DNA	Primary neurons	PEI-DNA-TAT improves the cellular uptake of gene vectors and enhances the gene transfection efficiency of primary neurons up to 14-fold [90]
